# Can a noisy venue be bad for comedy?

**DOI:** 10.1371/journal.pone.0332911

**Published:** 2025-09-19

**Authors:** Drew Gorenz, Norbert Schwarz

**Affiliations:** 1 Department of Psychology, University of Southern California, Los Angeles, California, United States of America; 2 Mind & Society Center, University of Southern California, Los Angeles, California, United States of America; 3 Marshall School of Business, University of Southern California, Los Angeles, California, United States of America; University of Melbourne, AUSTRALIA

## Abstract

People hear jokes live and pre-recorded in a variety of settings, from comedy clubs, bars, outdoor venues, cafes, to their own home or car. While a lot of research has analyzed the significance of the content of jokes, we know less about the significance of the setting one hears them in. Some settings can have interfering background noise or poor acoustics, reducing an audience’s ease of processing heard jokes. Would this affect how funny the jokes seem? Two experiments with audio clips of stand-up comedy performances show that participants found jokes less funny when background noise interfered with their listening.

## Introduction

People hear comedy live and pre-recorded in many contexts, from comedy clubs, bars, outdoor venues, and cafes to their own home or car [1]. While humor research has analyzed the significance of the content of jokes [2,3], we know less about the significance of the setting in which one hears them. Given that most modern comedy—including stand-up, improv, sketch shows, and comedic films or television—relies heavily on audio delivery, any factor that affects how easily listeners can process that audio may shape their experience of humor.

At live performances, the venue comedians perform at can greatly influence the audience’s ease of processing their jokes. While professional comedians may have access to well-managed comedy clubs or concert venues with great acoustics, amateur comedians often have to perform wherever they get an opportunity. Especially at the beginning of their careers, when they most need to build a following, comedians frequently perform at open mic events where the background noise of bars and cafes is poorly controlled, customers simultaneously pursue other activities, and acoustics are an afterthought. When consuming pre-recorded comedy, the settings in which audiences listen to jokes may matter as much as the setting in which they were recorded and the quality of the audio equipment used. People often listen to recordings in settings where background noise or poor sound quality can interfere with their processing. Commuters may listen to a comedy recording or podcast while traffic noise competes with the recording, or employees may watch comedy videos on their lunch break, while coworkers eat and talk nearby. Research in other domains suggests that such noisy settings may impair humor appreciation even when the audience fully understands the content.

For example, Newman and Schwarz [4] presented interviews from the radio show Science Friday in different sound qualities. Listeners found the interviewed researchers more likable and intelligent, and their research more compelling, when the sound mirrored the quality of a good studio line than when it mirrored the quality of a mobile phone connection. Similarly, Bild and colleagues [5] found that poor audio quality undermined the credibility of eyewitnesses and the impact of their testimony in virtual court settings. These adverse effects were not due to a lack of comprehension—participants understood the substantive points but were less persuaded by the communicator and the message when audio quality was poor. These observations are consistent with metacognitive research into processing fluency, that is, the subjective experiences of the ease or difficulty of mental processes (for reviews, see [6,7]). However, comedy serves a different purpose than educational content or testimony; its goal is to entertain rather than inform. It therefore remains unclear whether the adverse effects of low audio fluency extend to humor appreciation and the evaluation of comedians and their jokes.

Most humor theories highlight incongruity as a key element of funniness: the delivery of a joke sets up an expectation that is violated and eventually resolved by the audience (for reviews, see [8,9]). Any of these components—the expectation, its violation, and/or its resolution—may seem less compelling when processing feels difficult, thus impairing the appreciation of a joke. Compatible with this expectation, Topolinski [10] found that text-based jokes were rated as funnier when presented in easy-to-read fonts or when punchline elements were repeated, thus facilitating easy processing. A limitation of this research is that the jokes were all text-based and resembled difficult riddles (e.g., translated from German, “What were the last words of a vampire?” “Dawn!”). Not all comedy challenges the audience to generate an explicit resolution to a riddle before revealing the answer. Some comedy merely recounts an “odd” situation or a surprising observation about society. The lack of a cognitive challenge in this latter type of comedy may make the resulting ease or difficulty in processing it less relevant to one’s appreciation of its humor. The popular types of comedy people pay to see (i.e., stand-up comedy, comedy TV shows) are dominated by the latter types of jokes and use a longer and richer format (e.g., voice inflections and timing).

To better understand the extent to which processing fluency affects humor, the present studies examine audio fluency and use more ecologically valid comedic materials with fewer cognitive demands on the audience. In other words, we test the impact of fluency on passively consumed stand-up comedy rather than comedic riddles requesting active generation of joke resolutions. Stand-up comedy offers an ideal test case: it is professionally crafted, delivered acoustically, and widely consumed. If fluent processing enhances humor appreciation generally, then conditions that impair auditory clarity—such as ambient noise or poor sound quality—should reduce the funniness of the performance.

### Present studies

We conducted two experiments to test the influence of a noisy or quiet setting on humor appreciation. In the quiet setting condition, participants listened to the original recordings of stand-up comedians recounting odd personal experiences and noting their surprising observations about society. These recordings included some baseline audience noise in the form of laughter but were otherwise clear and easy to process. In the noisy-setting condition, the same audio clips were altered by adding the background sounds of a noisy cafe. Cafes are a common venue for open mic events that comedians and musicians may perform at, especially at the beginning of their careers. Of key interest is whether noisy background acoustics impair humor appreciation, as may be expected based on the fluency research discussed above [4,5,10]. Experiment 1 was embedded in a larger data collection, and Experiment 2 is a stand-alone replication. The results of both experiments converge.

## Experiment 1

### Methods

The pre-registration for this study and all supplementary materials and data are available at our OSF repository (https://osf.io/td49x/). Analyses were conducted in R [11].

### Design

We asked participants to listen to recordings of two different stand-up comedians performing in a cafe and to evaluate each performance. Participants heard one comedian with the added background sounds of a noisy cafe (low audio fluency condition) and the other in the original recording (high audio fluency condition). We counterbalanced the order of comedians and their assignment to audio fluency conditions across participants to control for the influence of comedian and judgment order on participants’ humor ratings. This results in a 2 (audio fluency: high (unedited clip) vs. low (noisy cafe audio added)) x 2 (comedian: A vs. B) x 2 (fluency order: high fluency first vs. low fluency first) x 2 (comedian order: comedian A first vs. comedian B first) mixed design with fluency and comedian as within-subjects repeated factors and comedian order and fluency order as between-subjects factors. Additional information about the design and number of participants per condition for each experiment is in the supplementary materials. After participants heard both recordings, they evaluated the stand-up comedians and their jokes.

### Participants

We recruited N = 274 students from our university’s psychology subject pool (68% female, *M*_age_ = 20.3, English first language 83%, 13% international students, slightly liberal **M* *= −1.52 on a scale from −4 (*Very Liberal)* to 4 (*Very Conservative*)). Following our preregistration, we excluded 60 participants who either indicated they were not in a setting to comfortably hear audio (n = 12), recognized either comedian (n = 6), or failed the audio fluency task’s content recognition check (n = 42), described below (final N = 214). Participants were no more likely to miss the content recognition question in the low fluency condition (McNemar’s *X*^*2*^ = 2.29, *p* = .13). Participants were recruited from 2/22/2022–4/18/2022.

### Materials & procedure

The experiment was conducted online as part of a larger data collection that included the norming of celebrity jokes for a later study. Participants rated the funniness of those stimuli before listening to the audio clips of the present experiment. Given that the preceding norming stimuli were identical for all participants and participants were randomly assigned to the subsequent experimental conditions, we have no theoretical reason to believe that the norming task exerted a differential impact on the experimental findings. Experiment 2 avoids this concern.

Participants listened to two stand-up comedy clips. We selected approximately one-minute-long segments from two comedians. To create the low fluency version of each comedy clip, we added bustling cafe noise (found on YouTube) using Garageband software. The added sound mirrored what one might hear in a busy cafe, such as indistinct chatter, the noise of people moving around, dishes clanging, and coffee beans grinding. For the high fluency version of the same comedy clips, we left the original recordings unedited. The original recordings included some baseline audience noise in the form of laughter but were otherwise clear and easy to process. The audio stimuli we used are available in our supplementary materials. Participants listened to both clips and then evaluated each comedian on humor and several other measures (all questions are listed in the supplementary materials).

Finally, we asked participants (1) to tell us what they thought the study was about and what they thought the hypothesis of the study was; (2) whether they had heard either stand-up comedian’s jokes before today; (3) two multiple-choice content recognition questions to ensure they had been paying attention to the audio stimuli. Additional questions followed that are unrelated to the current research issues. In addition, participants reported their comedy-seeking behavior, sex, age, political leaning, native language, and nationality. Comedy-seeking was included as an indicator of both interest in and familiarity with comedy. Familiarity with typical exemplars of an art form facilitates processing of other exemplars of the same art form, which increases aesthetic appreciation [6]. On the other hand, expertise in an art form can also be associated with lower reliance on fluency experiences and higher reliance on more substantive evaluation criteria [e.g., 12–14]. Political leaning was included because political orientation can influence responses to humor, especially when comedy engages with sensitive social or cultural issues [15,16]. After answering all questions pertaining to this experiment, participants completed a few separate tasks as part of a data collection for an unrelated study. All materials were presented via Qualtrics.

### Ethical considerations

The study was approved by our university’s Institutional Review Board (UP-21–01050; Date: 1/6/2022). The data were collected from students via an online questionnaire. Participants consented by checking a box before starting the study.

### Results

Participants found the comedian less funny when the recording mirrored the acoustics of a noisy cafe. As pre-registered, we tested whether the same jokes seem funnier when they are easier to process (1) by assessing how many participants chose the high fluency comedian as funnier and (2) by comparing participants’ mean funniness ratings of the high fluency comedian to the low fluency comedian. We ran two-tailed binomial tests for the choice measures and paired, two-tailed t-tests for ratings.

A two-tailed binomial test showed that a majority of participants chose the high fluency comedian as funnier (66.4%), which was significantly above chance (*p* < .001, 95% CI = [.60,.73]; Cohen’s *g* = 0.16). In contrast, only 33.6% chose the low fluency comedian (95% CI = [.27,.40]). Participants showed a small, but statistically insignificant preference for one comedian over the other (*M*_*Comedian A*_ = 56.5%, 95% CI = [.50,.63]; *M*_*Comedian B*_ = 43.5%, 95% CI = [.37,.50]); *p*_*binomial test*_ = .065, Cohen’s *g* = 0.07). However, the influence of audio fluency held for both comedians and was sufficient to reverse the majority preference on this dichotomous choice measure: whereas 73.3% preferred Comedian A when his performance was easier to process (95% CI = [.64,.81]; *p*_*binomial test*_ < .001, Cohen’s *g* = 0.23), 59.6% preferred Comedian B when his performance was easier to process (95% CI = [.50,.69]; *p*_*binomial test*_ = .055, Cohen’s *g* = 0.10).

This preference for the more fluently presented comedian held across the counterbalancing conditions, and we did not observe interactions between experimental and counterbalancing conditions (see supplementary materials). The positive effect of audio fluency on humor ratings emerged regardless of whether we asked participants to rate each “comedian” (*t*_*paired*_(213) = 5.79, *p* < .001, *d*_*rm*_ = 0.46) or each comedian’s “jokes” (*t*_*paired*_(213) = 4.63, *p* < .001, *d*_*rm*_ = 0.39). The differences in humor ratings and preferences remain statistically significant with a post-hoc Bonferroni correction for the multiple analyses.

**Fig 1:**
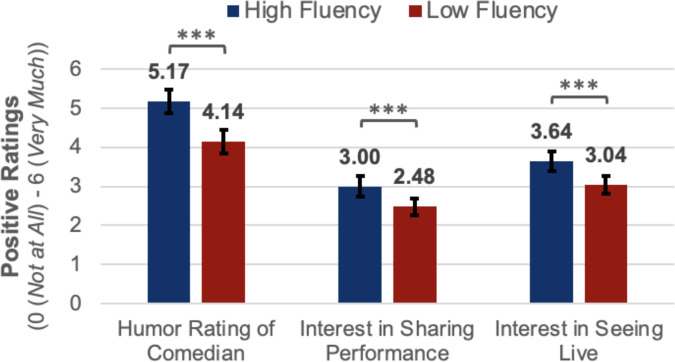
Positive ratings by fluency condition. Error bars represent 95% confidence intervals for mean ratings of stand-up comedy clips. Two-tailed, paired t-test results: * *p* < .05; ** **p* *< .01; *** **p* *< .001.

Participants were also more interested in seeing the higher fluency comedian live (*M*_*High Fluency*_* *= 3.64, 95% CI = [3.38, 3.89]; *M*_*Low Fluency*_* *= 3.04, 95% CI = [2.82, 3.27]; *t*(213) = 4.69, *p* < .001, *d*_*rm*_ = 0.33) and sharing his performance with others (*M*_*High Fluency*_* *= 3.00, 95% CI = [2.73, 3.26]; *M*_*Low Fluency*_* *= 2.48, 95% CI = [2.26, 2.69]; *t*_*paired*_(213) = 4.66, *p* < .001, *d*_*rm*_ = 0.28) (see Fig 1). A greater percentage of participants also indicated they would rather hear more jokes from the higher fluency comedian (*M*_*High Fluency*_* *= 66.4%, 95% CI = [.60,.73]; *p*_*binomial test*_ < .001, Cohen’s *g* = 0.16) than the lower fluency comedian (*M*_*Low Fluency*_* *= 33.6%, 95% CI = [.27,.40]). When prompted whether they would like to receive a link to each comedian’s full performance, a greater percentage of participants requested the link to the full performance of the high fluency comedian (*M* = 39.3%) than the low fluency comedian (*M* = 27.6%, *t*_*paired*_(213) = 3.22, *p* = .001, *d*_*rm*_ = 0.25). These mean differences remain statistically significant with a post-hoc Bonferroni correction for the four analyses.

### Discussion

Our experiment shows that poor audio fluency (in the form of cafe background noise) can decrease people’s appreciation of a joke’s humor. We saw this pattern in participants’ choices and ratings. They judged the same joke content and comedian as less funny when they heard it through a recording that mirrored the acoustics of a noisy cafe. Lower audio fluency also led to decreased engagement with the comedy on behavioral measures (requesting a link to the full performance) and attitude metrics.

One limitation of Experiment 1 is that we did not measure whether subjective reports of audio fluency differed between conditions, although intuitively, adding background noise should reduce processing fluency. In Experiment 2, we included a manipulation check to confirm that our audio fluency manipulation reduced participants’ subjective ease of processing as we assumed. As noted, Experiment 1 was also embedded in a larger data collection that included other questions about humor. This raises the possibility of unobserved contextual influences. To eliminate this concern, in Experiment 2, we conducted a stand-alone replication without preceding items.

## Experiment 2

### Methods

The pre-registration for this study and all supplementary materials and data are available at our OSF repository (https://osf.io/td49x/). Analyses were conducted in R [11].

### Design

As in Experiment 1, participants were randomly assigned to counterbalanced conditions and heard a recording of one comedian in low audio fluency and another in high audio fluency. After hearing both recordings, participants evaluated the stand-up comedians and their jokes.

### Participants

We recruited N = 143 students from our university’s psychology subject pool (62% female, *M*_age_ = 20.4, English first language 73%, 17% international students, slightly liberal **M* *= −1.03 on a scale from −4 (*Very Liberal)* to 4 (*Very Conservative*)). Following pre-registered criteria, we excluded 35 participants who either indicated they were not in a setting to comfortably hear audio (n = 6), they recognized one of the comedians (n = 6), or failed the audio fluency task’s content recognition check (n = 23). This leaves N = 108 for analyses. Participants were no more likely to miss the content recognition question in the low fluency than in the high fluency condition (McNemar’s *X*^*2*^ = 0.20, *p* = .65). Participants were recruited from 3/20/2023–3/30/2023.

### Materials & procedure

We used the same materials and procedures as in Experiment 1, with three small exceptions: 1) there were no other items preceding this experiment; 2) we reduced the number of redundant dependent measures (see supplementary materials for details); and 3) added an audio fluency manipulation check. After participants listened to both comedians’ recordings, they indicated which comedian they thought was funnier, how funny they found each comedian, and how interested they were in sharing a clip of each comedian with their friends (all reported on the same scales as in Experiment 1). Next, we asked participants to what extent the process of hearing each comedian’s comedy set was easy or difficult on a 100-point sliding scale (1 = *difficult*; 101 = *easy*). This single-item measure of fluency was adapted from Graf and colleagues [17], who validated this manipulation check by confirming its predictive value for several classic fluency effects.

In addition, participants were asked whether they had heard either stand-up comedian’s jokes before, and two multiple-choice content recognition questions to ensure they had paid attention to the audio stimuli. Participants also reported their general comedy-seeking behavior, sex, age, political leaning, native language, and nationality. All questions were presented via Qualtrics.

### Ethical considerations

The study was approved by our university’s Institutional Review Board (UP-21–01050; Date: 1/6/2022). The data were collected from students via an online questionnaire. Participants consented by checking a box before starting the study.

### Results

Participants listened to a high fluency recording (resembling a quiet cafe) of one comedian and a low fluency recording (resembling a bustling cafe) of another comedian. The manipulation check confirmed the intended difference in fluency. Participants rated the process of hearing the low audio fluency clip as more difficult (*M* = 46.1) than the high audio fluency clip (*M* = 83.6) (*t*_*paired*_(107) = 9.96, *p* < .001, *d*_*rm*_ = 1.44).

Replicating the analyses in Experiment 1, we ran two-tailed binomial tests for the choice measures and paired, two-tailed t-tests for ratings. A two-tailed binomial test showed that a majority of participants chose the high fluency comedian as funnier (69.4%), which was significantly above chance (*p* < .001, 95% CI = [.60,.78], Cohen’s *g* = 0.19). In contrast, only 30.6% chose the low fluency comedian (95% CI = [.22,.40]). Overall, Comedian A was, again, chosen as the funnier comedian more often than Comedian B (*M*_*Comedian A*_ = 66.7%, 95% CI = [.57,.75]; *M*_*Comedian B*_ = 33.3%, 95% CI = [.25,.43]; *p*_*binomial test*_ < .001, Cohen’s *g* = 0.17), for the main effect. However, the influence of audio fluency emerged for both comedians and was sufficient to reverse the majority preference on the dichotomous choice measure. Whereas 86.8% preferred Comedian A when his performance was easier to process (95% CI = [.75,.95]; *p*_*binomial test*_ < .001, Cohen’s *g* = 0.37), 52.7% preferred Comedian B when his performance was easier to process (95% CI = [.39,.66]; *p*_*binomial test*_ = .79, Cohen’s *g* = −0.03), although the latter difference was not significant.

**Fig 2:**
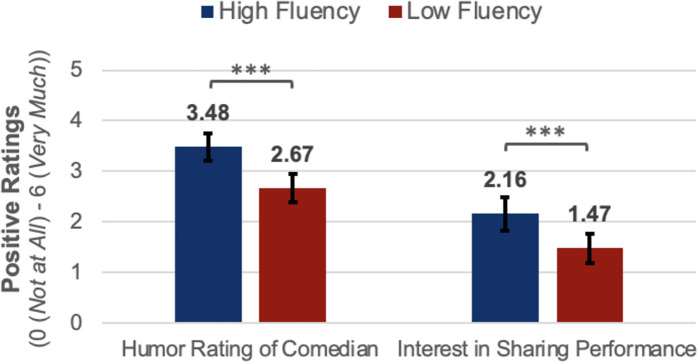
Positive ratings by fluency condition. Error bars represent 95% confidence intervals for mean ratings of stand-up comedy clips. Two-tailed, paired t-test results: * *p* < .05; ** **p* *< .01; *** **p* *< .001.

We did not observe any interactions between experimental and counterbalancing conditions (see supplementary materials). The positive effect of audio fluency emerged regardless of whether we analyzed participants’ choice preference or their ratings (*M*_*High Fluency*_* *= 3.48; *M*_*Low Fluency*_* *= 2.67) (*t*_*paired*_(107) = 4.58, *p* < .001; *d*_*rm*_ = 0.56) of each comedian. Participants were more interested in sharing a link to the comedian they heard in the high fluency condition (*M* = 2.16) than in the low fluency condition (*M* = 1.47) (*t*_*paired*_(107) = 3.88, *p* < .001; *d*_*rm*_ = 0.41) (see Fig 2).

### Discussion

Study 2 replicated Study 1’s findings that poor audio fluency (in the form of continuous background cafe noise) can decrease people’s appreciation of a joke’s humor. We saw this pattern across people’s choices and ratings. People judged the same joke content and comedian as less funny when they heard it through a recording with added background audio that mirrored the acoustics of a loud cafe.

## General discussion

The converging results of both experiments bear on the theoretical and applied aspects of humor appreciation. On the theoretical side, they shed light on the role of processing fluency in humor appreciation. On the applied side, they bear on the influence of setting on the enjoyment of comedy and comedians’ success.

## Fluency and humor appreciation

Any mental operation, from perception to judgment and retrieval, can feel easy or difficult. The experience of ease or difficulty, commonly referred to as high or low processing fluency, has been found to influence a wide range of judgments, from assessments of one’s knowledge [18] and learning [19] to judgments of beauty [6], truth [20], and trust [21], among others (for reviews, see [7,22,23]). Extending the exploration of fluency effects to humor appreciation, Topolinski [10] found that text-only, riddle jokes seem funnier when they are easier to process due to better readability of the print font or the repetition of some elements. Using audio recordings of stand-up comedy, the present experiments show that spoken jokes are judged funnier when they are easy rather than hard to process auditorily. The influence of processing fluency was sufficiently pronounced to result in reversals of the majority preference. One potential limitation is that audio fluency may have a reduced impact on humor appreciation when combined with visual elements, as would be the case in live performances or audio-visual recordings. Although low audio quality hurts the evaluation of video recordings of scientific talks [4], eyewitness testimony [5], and job interviews [24], systematic comparisons of the impact of audio quality in the context of different visual cues have not been conducted. Future studies may fruitfully investigate whether poor audio fluency detracts from audio-video comedy to a similar extent as audio-only comedy and whether closed captions moderate the effect by facilitating easier processing.

The observed adverse impact of low auditory fluency converges with findings in other judgment domains. Low auditory fluency impairs the evaluation of researchers and the credibility of their findings when conference talks are video-recorded in low audio quality or radio interviews sound like the researcher had a poor phone connection [4]. Similarly, poor audio undermines the credibility of witnesses in virtual court proceedings [5] and the appeal of job candidates in video interviews [24], as does a difficult-to-understand accent [25].

Importantly, a wide range of variables can make processing easy or difficult. They include person variables, such as the perceiver’s knowledge and exposure history; stimulus variables, such as the coherence or complexity of a message; presentation variables, such as the readability of a print font or the clarity of audio or a speaker’s accent; and context variables, from distraction to contextual influences on concept accessibility [23]. All of these variables have been found to exert parallel effects on evaluative judgment, indicating that the crucial driver of fluency effects is the subjective experience of ease or difficulty rather than the specific variable that induces the experience (for reviews, see [6,22,23]). We assume that this will also hold for humor appreciation, which provides a promising avenue for the systematic exploration of variables that facilitate or impair the enjoyment of comedy.

## Venue selection

The key element of stand-up comedy is the comedian’s jokes. Unfortunately, many venue characteristics have the potential to impair the audience’s ability to easily process the jokes. People hear comedy live and pre-recorded in a variety of contexts, from comedy clubs, bars, outdoor venues, cafes, to their own home or car [1]. As our experiments illustrate, contextual noise that impairs processing also impairs the audience’s appreciation of the performance. Hence, comedians would be well-advised to be picky about the acoustics and sound quality of the venues they choose to perform in, and comedy consumers should not just consider “who” they will hear perform, but also “where.” While the richer context cues offered by a live performance may attenuate the adverse effects of low audio fluency observed in the present experiments, studies on the evaluation of video-recorded conference talks [4], eyewitness testimony [5], and job interviews [24] suggest that they are unlikely to overcome them. Future research may shed light on this issue by taking advantage of natural variations in audio quality at live performances, where some seats enjoy better acoustics than others.
